# Pulmonary Arterial Hypertension Induces a Distinct Signature of Circulating Metabolites

**DOI:** 10.3390/jcm9010217

**Published:** 2020-01-14

**Authors:** Ruslan Rafikov, Dawn K Coletta, Lawrence J. Mandarino, Olga Rafikova

**Affiliations:** Department of Medicine, Division of Endocrinology, University of Arizona College of Medicine, Tucson, AZ 85721, USA; ruslanrafikov@deptofmed.arizona.edu (R.R.); dcoletta@deptofmed.arizona.edu (D.K.C.); mandarino@deptofmed.arizona.edu (L.J.M.)

**Keywords:** pulmonary arterial hypertension, diabetes, metabolites, biomarkers

Pulmonary arterial hypertension (PAH) is an incurable, progressive disorder, and the early diagnosis and treatment of PAH are associated with increased survival [1,2]. However, because of the asymptomatic nature of PAH development, there are severe delays in PAH diagnosis. In most cases, therapy only starts at an advanced stage of the disease. Thus, there is a critical need to improve the diagnostic approaches, for example, by using markers of PAH that can be detected in easily obtained specimens like plasma or urine [3]. Our previous study showed that in the monocrotaline-treated rat model, metabolic reprogramming occurs early in the development of the disease and foreshadows pathophysiological changes [4]. Thus, assessing the profile of circulating metabolites could allow the identification of PAH patients earlier, before the condition has progressed sufficiently to enter an advanced irreversible stage.

Several previous reports have already confirmed a significant difference in the metabolic profile of healthy controls and PAH animals and patients [4,5,6,7]. However, given that the average age of PAH patients has increased [8], there is a strong need to diagnose PAH in older people that are not entirely “healthy.” In this study, we compared the plasma metabolic profiles of well-phenotyped patients with idiopathic PAH (IPAH) obtained from the Pulmonary Hypertension Breakthrough Initiative (PHBI) with two other patient cohorts—patients with type 2 diabetes mellitus (DM; University of Arizona (UA) Center for Disparities in Diabetes, Obesity, and Metabolism) and patients with left heart diseases (Heart; UA biobank). The incidence of non-PAH cardiovascular diseases and type 2 diabetes significantly increases with age, especially in people over 40–45 years old [9,10]. Furthermore, these conditions are associated with severe alterations in metabolism that could mask the changes associated with metabolic reprogramming in PAH [11,12]. Therefore, the goal of the study was to evaluate whether the metabolic profile of PAH patients is distinct compared to these patients and could be used to identify PAH when compared to people who have one of these diseases. 

The patients’ ages were (mean ± standard deviation (SD)) 41.1 ± 15.7, 41.8 ± 13.7, and 50.2 ± 10.9, and the sex ratios of female/male (%) were 81.8/18.1, 83.3/16.7, and 72.7/27.3 for PAH (*n* = 11), DM (*n* = 12), and heart (*n* = 11), respectively. All of the PAH patients were receiving PAH therapy. No patients from the PAH cohort had DM; one patient had valvular left ventricular (LV) heart disease. The targeted metabolomics approach was used to quantify the primary plasma metabolites, including carbohydrates, amino acids, and nucleotides, by gas-chromatography time of flight (GC-TOF) mass spectrometry (UC Davis NIH West Coast Metabolomics). Two runs of profiling were acquired separately, PAH versus DM and PAH versus heart. In the DM/PAH run, 84 metabolites (out of 172 analyzed) were significantly altered (*p* < 0.05). A comparison of the PAH and heart groups showed 76 significantly changed metabolites among the 158 that were identified. Heat maps (Figure 1A,B) show a characteristic clustering of the PAH group, distinguishing them from DM and heart patients. The principal component analysis (PCA) of significantly changed metabolites (Figure 1C,D) showed a clear separation of the PAH sub-population from DM and heart patients. Thus, the plasma metabolomics profile of patients with PAH is distinctly different compared with diabetic patients or patients with left heart diseases. 

Moreover, we have identified 32 unique metabolites that are significantly altered in PAH compared with either control group and could be used as a fingerprint for PAH (Table 1). These unique metabolites could be classified into a few groups. The primary group consists of mitochondrial-derived metabolites, including tricarboxylic acid (TCA) metabolites and their derivatives, which have been reported as significantly altered in PAH subjects compared with healthy controls [13], and may indicate undergoing anaplerotic reactions. Thus, oxalic acid, the product of oxaloacetate decomposition, showed a ~20 fold increase in PAH compared with either control cohort. The next largest group consisted of carbohydrates that could become elevated as a result of the glycolytic shift associated with PAH pathogenesis. Vascular remodeling in PAH could be the main reason for a decreased level of circulating amino acids, the primary building blocks of proteins that are highly consumed by proliferating cells. Elevated levels of plasma myo-inositol and its derivatives strongly correlate with our recently published animal data, demonstrating that myo-inositol could play an important role in proliferative signaling in PAH [14]. The last two groups contain metabolites associated with PAH-mediated damage, or with the altered gut microbiome, which may also be involved in PAH pathogenesis [15]. 

Although the analysis of PAH samples versus DM or heart cohorts was assessed in two different runs, we found a very robust reproducibility of the metabolic data (Figure 2). Therefore, we were able to combine two experiments using the PAH group as a reference point and compare all three patients’ cohorts together (Figure 3). Moreover, we undertook metabolite optimization and identified the minimal number of metabolites sufficient for a significant separation of the PAH group from DM and heart. In Figure 3, only eleven metabolites were used to distinguish the PAH samples from the other patients. This analysis indicates that PAH patients metabolically are well resolved from both diseases. Thus, this panel of eleven metabolites (oxalic acid, pseudouridine, gluconic acid, fumaric acid, uridine diphosphate (UDP)-glucuronic acid, aconitic acid, erythritol, 2-deoxytetronic acid, glutamic acid, inorganic phosphate, and 2-hydroxyglutaric acid) could be used for the pre-screening of patients to identify PAH at the early asymptomatic stage, or could help to minimize the time for PAH diagnosis after the onset of the initial symptoms, reported to be currently 47.1 ± 34.2 months [16].

There are limitations to this study. Thus, PAH-specific therapies or the size of the patient cohort could affect the outcome of metabolic profiling. Therefore, the larger cohort of patients and an analysis of the different PAH cohorts is required in order to confirm our conclusions and to estimate the prognostic value of metabolic profiling. Moreover, the future assessment of the other pulmonary hypertension (PH) World Health Organization (WHO) groups would estimate whether the discovered metabolic fingerprint could be applied toward the patients with different types of PH. Thus, it has been recently reported that not only PAH but other types of PH, including exercise-induced PH and chronic thromboembolic pulmonary hypertension (CTEPH), have a metabolomic pattern that is different compared with the control subjects [13,17]. However, while the PAH cohorts evaluated in these studies showed changes similar to our findings, such as increased levels of TCA cycle metabolites (fumarate, citrate, and malate), glycolysis intermediates (lactate and pyruvate), nucleosides (pseudouridine and urate), and ketone bodies (butyric acid derivatives), either exercise-induced PH or CTEPH produced less prominent changes and showed different types of metabolic alterations. These results suggest that an increase in the pulmonary pressure occurring not because of the over-proliferative processes in the pulmonary vasculature, but secondary to other triggers (WHO groups 2–5) or mild/undeveloped forms of PH (exercise-induced PH), could require individual metabolic profiling.

The heterogeneous nature of control cohorts (DM and heart) should also be considered, although both control groups were found to be metabolically more homogeneous than the IPAH cohort (Figure 1C,D). We also understand that the conditions chosen are just a fraction of the diseases that could present in the general population. In the future, the same analysis should be extended to include other pathologies that metabolically can overlap with PAH patients, such as cancer, lung diseases, and systemic hypertension. Nevertheless, we believe that these findings will spark a discussion in the field on the potential value of metabolic profiling as a new diagnostic tool, and provide subsequent research with a specific set of preselected metabolites that could serve as a fingerprint of PAH. Furthermore, these results may highlight the potential value of the particular metabolites in dissecting the pathogenesis of PAH. 

## Figures and Tables

**Figure 1:**
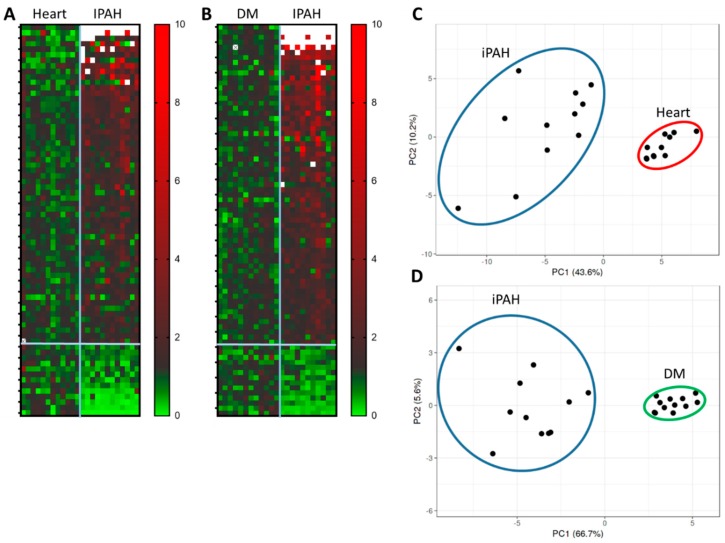
Metabolic profiling of plasma of pulmonary arterial hypertension (PAH) patients (*n* = 11), and comparison with diabetes mellitus (DM; *n* = 12) and left heart disease (heart; *n* = 11) patients. Our data indicate significantly (*p* < 0.05) altered metabolites in PAH vs. heart and PAH vs. DM analysis. (**A**,**B**) Heat maps and (**C**,**D**) principal component analysis (PCA) show the clustering of the PAH group that could differentiate PAH patients from DM and heart by circulating metabolites. Ellipses indicate the area of a 0.95 probability that the metabolic profiling of the patient from the same group will be inside an ellipse.

**Figure 2:**
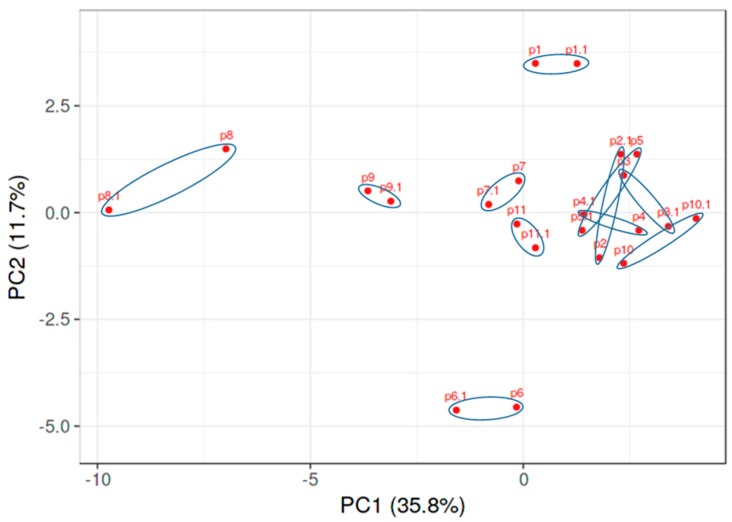
High reproducibility of PAH metabolic profile obtained from two independent experiments. An analysis of two independent runs of plasma metabolites from PAH patients (*n* = 11). Circles indicate the same patient in two experiments.

**Figure 3:**
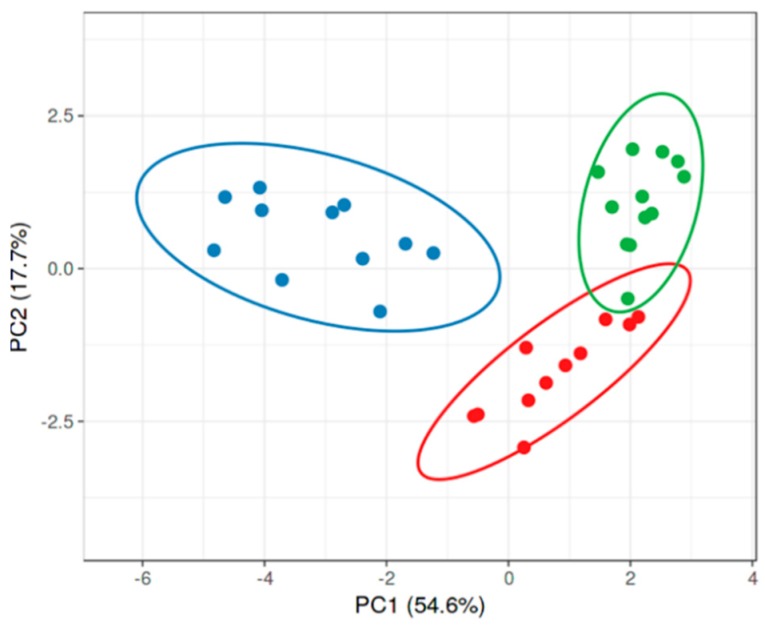
Optimization of the metabolic platform to distinct idiopathic PAH (IPAH) patients vs. DM or heart cohorts. The identified profile of eleven metabolites was sufficient to provide a significant separation of PAH patients (*n* = 11), from either patients with diabetes mellitus (DM; *n* = 12) or patients with left heart disease (heart; *n* = 11). Ellipses indicate the area of 0.95 probability that the metabolic profiling of the patient from the same group will be inside an ellipse.

**Table 1:** Metabolites found to be significantly different in PAH cohort vs. heart or DM cohorts.

Metabolites	Fold Heart	*p*-Value	Fold DM	*p*-Value
oxalic acid	45.23	4.35 × 10^−5^	82.10	2.24 × 10^−8^
aminomalonate	3.00	6.52 × 10^−5^	2.75	6.12 × 10^−3^
pseudo uridine	2.62	2.31 × 10^−4^	3.07	8.86 × 10^−5^
gluconic acid	2.35	6.25 × 10^−8^	1.55	1.33 × 10^−2^
isothreonic acid	2.05	1.31 × 10^−5^	2.09	9.85 × 10^−4^
4-hydroxyphenylacetic acid	2.02	1.57 × 10^−2^	2.24	2.77 × 10^−3^
erythritol	2.01	3.16 × 10^−3^	2.83	1.56 × 10^−3^
uric acid	1.99	2.28 × 10^−3^	1.79	4.85 × 10^−2^
UDP-glucuronic acid	1.98	4.45 × 10^−3^	4.49	2.77 × 10^−4^
fumaric acid	1.95	3.81 × 10^−5^	1.37	1.16 × 10^−2^
focuse	1.86	1.68 × 10^−3^	1.68	3.40 × 10^−3^
aconitic acid	1.86	5.21 × 10^−5^	3.14	1.68 × 10^−9^
2-deoxytetronic acid	1.78	1.36 × 10^−2^	2.17	7.04 × 10^−5^
pantothenic acid	1.75	7.33 × 10^−3^	1.69	8.60 × 10^−4^
indole-3-acetate	1.59	9.92 × 10^−3^	2.06	1.16 × 10^−3^
myo-inositol	1.50	2.52 × 10^−2^	1.65	2.32 × 10^−2^
2-hydroxyvaleric acid	1.48	3.27 × 10^−2^	2.12	5.16 × 10^−3^
citric acid	1.48	2.97 × 10^−2^	2.01	1.81 × 10^−4^
ribonic acid	1.48	6.03 × 10^−4^	2.61	5.75 × 10^−4^
glycine	1.44	5.18 × 10^−2^	1.41	4.17 × 10^−2^
glutamic acid	1.40	4.91 × 10^−2^	0.68	3.59 × 10^−2^
creatinine	1.38	1.55 × 10^−2^	1.58	8.82 × 10^−4^
glucuronic acid	1.37	8.21 × 10^−4^	2.66	1.77 × 10^−4^
phosphate	1.28	4.56 × 10^−2^	4.54	2.09 × 10^−11^
indole-3-lactate	1.26	3.59 × 10^−2^	1.85	4.37 × 10^−6^
urea	1.21	2.91 × 10^−2^	1.47	2.49 × 10^−2^
2-hydroxyglutaric acid	0.78	2.88 × 10^−2^	1.55	2.80 × 10^−4^
tryptophan	0.76	4.66 × 10^−3^	0.51	7.55 × 10^−8^
tyrosine	0.74	7.99 × 10^−3^	0.67	3.43 × 10^−5^
glutamine	0.73	4.99 × 10^−3^	0.71	2.97 × 10^−2^
lysine	0.72	4.38 × 10^−4^	0.56	2.20 × 10^−6^
histidine	0.51	1.34 × 10^−5^	0.45	8.65 × 10^−7^
